# Deacetylase Plus Bromodomain Inhibition Downregulates ERCC2 and Suppresses the Growth of Metastatic Colon Cancer Cells

**DOI:** 10.3390/cancers13061438

**Published:** 2021-03-22

**Authors:** Sabeeta Kapoor, Trace Gustafson, Mutian Zhang, Ying-Shiuan Chen, Jia Li, Nhung Nguyen, Jorge Enrique Tovar Perez, Wan Mohaiza Dashwood, Praveen Rajendran, Roderick H. Dashwood

**Affiliations:** 1 Center for Epigenetics & Disease Prevention, Texas A&M Health, Department of Translational Medical Sciences, Texas A&M College of Medicine, Houston, TX 77030, USA; sabeetak@tamu.edu (S.K.); tracegustafson@gmail.com (T.G.); zhang1991@exchange.tamu.edu (M.Z.); vickychen0409@gmail.com (Y.-S.C.); jiali@tamu.edu (J.L.); nhungtuan0909@tamu.edu (N.N.); jtovar@tamu.edu (J.E.T.P.); wdashwood@tamu.edu (W.M.D.); 2 Antibody & Biopharmaceuticals Core, Texas A&M Health, Houston, TX 77030, USA

**Keywords:** BET inhibition, colorectal cancer, DNA repair, epigenetic, HDAC inhibition, JQ1, nucleotide excision repair, polyposis in rat colon, PROTAC, sulforaphane

## Abstract

**Simple Summary:**

In cancer cells, the DNA repair response can be exploited as an ‘Achilles heel’ to trigger programmed cell death pathways and tumor elimination. Rather than involving ‘naked’ DNA, repair occurs in the context of histone and non-histone proteins in the vicinity of the damage. Drugs that target different epigenetic mechanisms can lead to the synergistic downregulation of critical DNA repair factors, including those associated with poor survival in colorectal cancer patients. Notably, normal colonic epithelial cells are more resistant than colon cancer cells to the epigenetic drug combinations. In the current investigation, cell-based assays and preclinical animal models reaffirmed the crosstalk between DNA repair and epigenetic regulatory mechanisms, and provided new avenues for precision oncology and cancer interception.

**Abstract:**

There is growing evidence that DNA repair factors have clinical value for cancer treatment. Nucleotide excision repair (NER) proteins, including excision repair cross-complementation group 2 (ERCC2), play a critical role in maintaining genome integrity. Here, we examined ERCC2 expression following epigenetic combination drug treatment. Attention was drawn to ERCC2 for three reasons. First, from online databases, colorectal cancer (CRC) patients exhibited significantly reduced survival when *ERCC2* was overexpressed in colon tumors. Second, *ERCC2* was the most highly downregulated RNA transcript in human colon cancer cells, plus *Ercc2* in rat tumors, after treatment with the histone deacetylase 3 (HDAC3) inhibitor sulforaphane (SFN) plus JQ1, which is an inhibitor of the bromodomain and extraterminal domain (BET) family. Third, as reported here, RNA-sequencing of polyposis in rat colon (Pirc) polyps following treatment of rats with JQ1 plus 6-methylsulfinylhexyl isothiocyanate (6-SFN) identified *Ercc2* as the most highly downregulated gene. The current work also defined promising second-generation epigenetic drug combinations with enhanced synergy and efficacy, especially in metastasis-lineage colon cancer cells cultured as 3D spheroids and xenografts. This investigation adds to the growing interest in combination approaches that target epigenetic ‘readers’, ‘writers’, and ‘erasers’ that are deregulated in cancer and other pathologies, providing new avenues for precision oncology and cancer interception.

## 1. Introduction

Many anticancer therapeutics, by design, induce DNA damage and activate cell death pathways in cancer cells [1]. However, the DNA damage can be circumvented via endogenous mechanisms that include base excision repair, mismatch repair, double-strand break repair, and nucleotide excision repair (NER) [2,3,4]. The latter pathway repairs DNA lesions produced by UV light, environmental agents such as polycyclic aromatic hydrocarbons (PAHs), and chemotherapy drugs that cause pyrimidine dimers, bulky covalent DNA adducts, and DNA cross-links, respectively [5,6,7]. Excision repair cross-complementing rodent repair deficiency complementation group 2/xeroderma pigmentosum group D (ERCC2/XPD) plays a crucial role in the etiology of colorectal cancer (CRC) [8]. As a component of the Transcription factor II human (TFIIH) complex, ERCC2 is necessary for transcription initiation, DNA damage recognition, and NER in eukaryotes [9]. Through its helicase activity in the TFIIH complex, ERCC2 facilitates DNA duplex opening at transcription start sites and DNA damage locations [10]. Several cancers overexpress ERCC2, resulting in poor response to chemotherapy or radiation therapy, as demonstrated in late-stage CRC [11]. Thus, the downregulation or inactivation of ERCC2 in CRC and in other malignancies is an attractive strategy for tumor-targeting and chemosensitization.

Synergistic anticancer activity was observed in pancreatic ductal adenocarcinoma when combining the pan-histone deacetylase (HDAC) inhibitor suberoylanilide hydroxamic acid (SAHA, Vorinostat) with JQ1, a bromodomain and extraterminal domain (BET) inhibitor [12]. Our previous report [13] showed that JQ1 plus sulforaphane (SFN), a histone deacetylase 3 (HDAC3) inhibitor, were highly synergistic and downregulated *ERCC2* in human CRC cells. Murine *Ercc2* also was downregulated in adenomatous tumors from the polyposis in rat colon (Pirc) model, coinciding with anticancer outcomes for JQ1 + SFN in vivo [13].

We sought to extend these findings by examining ‘second-generation’ combination agents, including a more potent HDAC inhibitor SFN analog, 6-methylsulfinylhexyl isothiocyanate (6-SFN) [14,15], and Proteolysis Targeting Chimeric (PROTAC)-based BET degraders. Promising leads were obtained for future clinical translation in CRC patients, with ERCC2 as a mechanistic target.

## 2. Results

### 2.1. 6-SFN + JQ1 Act Synergistically in Human Colon Cancer Cells and Suppress Colon Polyps In Vivo 

**Figure 1:**
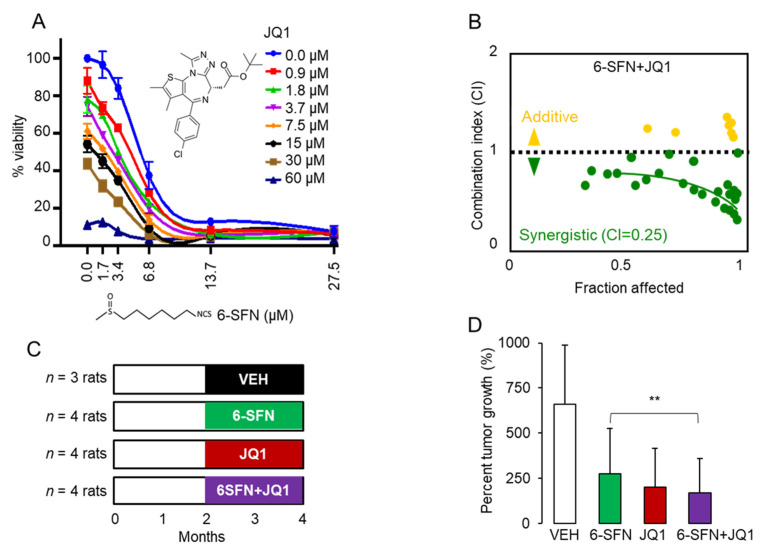
Inhibition by 6-SFN + JQ1 in human colon cancer cells and in the Pirc rat. (**A**) Viability of HCT116 human colon cancer cells treated with 6-SFN, JQ1, or 6-SFN + JQ1 over a range of concentrations for 48 h. Mean ± SE, *n* = 3 biological replicates. (**B**) Combination index (CI) data for HCT116 cells treated as in panel A; CI < 1.0 indicates synergy; the lowest CI value of 0.25 indicated highly synergistic. (**C**) Pirc males received corn oil (Vehicle, VEH), 6-methylsulfinylhexyl isothiocyanate (6-SFN) (10 mg/kg, *p.o.*), JQ1 (10 mg/kg, intraperitoneal (*i.p.*)), or 6-SFN + JQ1 twice a week for 7–8 weeks, as detailed in Materials and Methods. (**D**) Tumor growth inhibition by test agents vs. VEH, ** *p* < 0.01.

The viability of HCT116 human colon cancer cells was reduced markedly by 6-SFN and JQ1 (Figure 1A), whereas 6-SFN + JQ1 exhibited strong synergy, with a combination index (CI) of 0.25 (Figure 1B), similar to that reported for SFN + JQ1 in vitro [13]. Cell viability data were corroborated in colony formation assays (Figure S1). In vivo, Pirc males were treated with 6-SFN, JQ1, 6-SFN + JQ1, or vehicle (Figure 1C). At the end of the study, 6-SFN, JQ1, and 6-SFN + JQ1 suppressed colon tumor growth significantly (Figure 1D, ** *p* < 0.01). Although synergy was less evident, a longer duration of treatment than 2 months might favor such an outcome for 6-SFN + JQ1 in vivo.

### 2.2. Transcriptomics Prioritizes Ercc2 as a Key ‘Synergy/Cooperativity’ Gene in Pirc Colon Tumors

**Figure 2:**
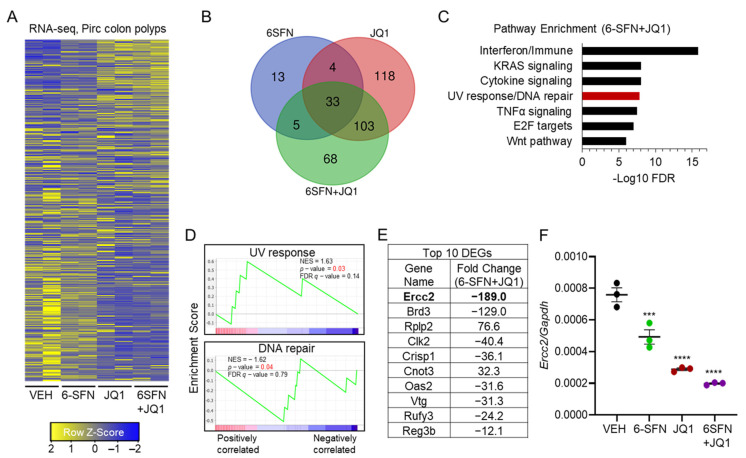
Transcriptomics prioritized *Ercc2* as the most highly altered gene by 6-SFN + JQ1 in Pirc colon polyps. (**A**) Heatmap of differentially expressed genes (DEGs) identified by RNA-seq analysis in Pirc colon tumors. Each column is a biological replicate (*n* = 2). Adjusted-*p* < 0.05, |FC| > 1. (**B**) Venn diagrams of DEGs in 6-SFN, JQ1, and 6-SFN + JQ1 groups vs. VEH controls; 6-SFN + JQ1-specific DEGs (*n* = 68) were treated as ‘cooperativity/synergy’ candidates for further analysis. (**C**) Pathway enrichment analysis for 6-SFN + JQ1 DEGs. (**D**) Gene set enrichment analysis for UV response and DNA repair pathways. (**E**) Top 10 most highly altered 6-SFN + JQ1-specific genes, ranked by absolute fold change (nine downregulated and one upregulated). (**F**) *Ercc2* mRNA expression in Pirc colon polyps by RT-qPCR, normalized to *Gapdh*; *** *p* < 0.001, **** *p* < 0.0001.

Paired colon polyps from groups in the Pirc study (Figure 1C,D) were subjected to RNA-seq and RT-qPCR analyses. The ‘heatmap’ of RNA-seq data (Figure 2A) prioritized 68 combination-specific ‘cooperativity/synergy’ candidates among the 209 total differentially expressed genes (DEGs) in the 6-SFN + JQ1 group (green circle, Figure 2B). The top five pathways implicated after 6-SFN + JQ1 treatment included Immune, Kirsten Rat Sarcoma viral oncogene homolog (KRAS), cytokine signaling, DNA repair, and Tumor Necrosis Factor alpha (TNFα) signaling (Figure 2C), whereas pathway enrichment analysis confirmed that DNA damage and DNA repair genes had net enrichment scores (NES) of +1.63 and −1.62, respectively (Figure 2D). Notably, with a net change in expression of −189 vs. vehicle, *Ercc2* was the most highly altered gene following 6-SFN + JQ1 treatment (Figure 2E). Subsequent RT-qPCR experiments corroborated the significantly reduced expression of *Ercc2* in Pirc colon polyps (Figure 2F), in the relative order: vehicle > 6-SFN > JQ1 = 6-SFN + JQ1. 

### 2.3. ERCC2 Overexpression Is Associated with Reduced Survival in CRC Patients

**Figure 3:**
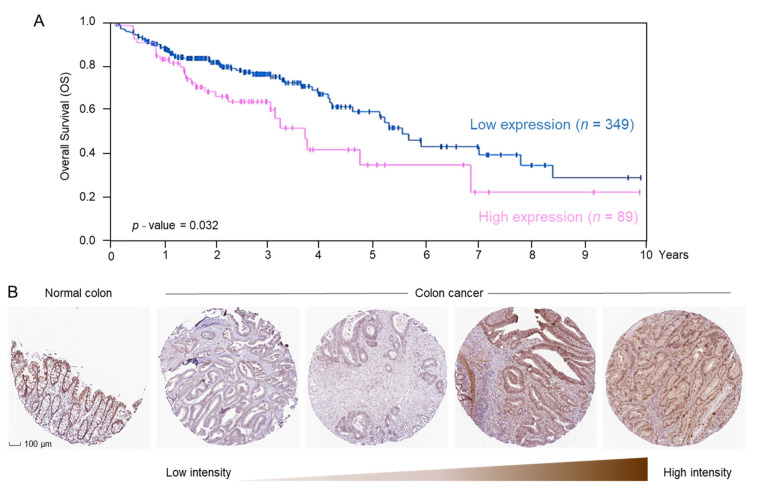
*ERCC2* overexpression is associated with reduced survival in CRC patients. (**A**) Overall survival (OS) in colorectal cancer (CRC) patients with high vs. low *ERCC2* mRNA expression in tumors from the Human protein atlas (https://www.proteinatlas.org/ENSG00000104884-ERCC2/pathology/colorectal+cancer/COAD, accessed on 18 September 2020) (**B**) Immunohistochemistry (IHC) images of ERCC2 protein expression in normal human colon tissue (https://www.proteinatlas.org/ENSG00000104884-ERCC2/tissue/colon#img, accessed on 18 September 2020) and in colon cancer (https://www.proteinatlas.org/ENSG00000104884-ERCC2/pathology/colorectal+cancer#img, accessed on 18 September 2020), from the Human Protein Atlas.

In CRC patient data obtained from the Human Protein Atlas, available from http://www.proteinatlas.org, accessed on 18 September 2020, *ERCC2* mRNA levels were associated with significantly reduced overall survival (OS, Figure 3A). Tissue microarrays revealed that ERCC2 protein was immunolocalized to the nuclear/membrane/cytoplasmic compartments (Figure 3B). A range of ERCC2 protein expression was observed, from low or undetectable to strongly positive in adenocarcinomas from CRC patients. Normal colon had medium ERCC2 protein expression and a distinct colonic crypt architecture (Figure 3B).

### 2.4. 6-SFN + JQ1 Co-Treatment Downregulates ERCC2 in Human Colon Cancer Cells

**Figure 4:**
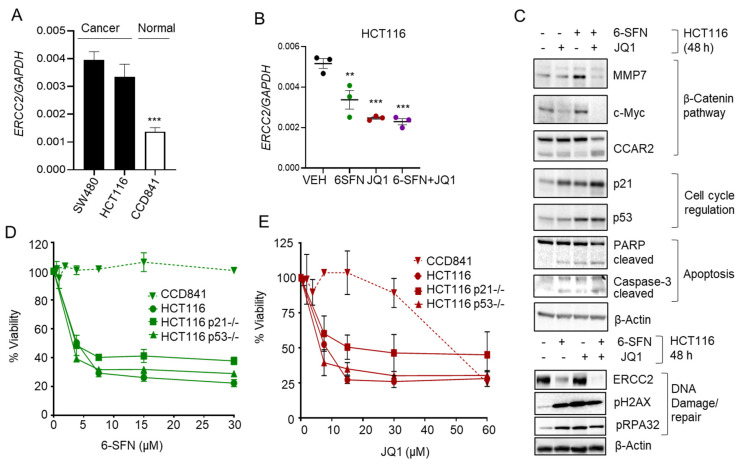
6-SFN + JQ1 co-treatment downregulates ERCC2 in human colon cancer cells. (**A**) *ERCC2* expression analyzed by RT-qPCR in tumor (SW480, HCT116) and normal (CCD841) cells, and (**B**) treatment of HCT116 cells with drugs for 48 h; ** *p* < 0.01, *** *p* < 0.001. (**C**) Immunoblot analysis of cell lysates treated as in B; β-Actin, loading control. (**D**,**E**) Viability of CCD841 normal, HCT116, HCT116 p21^−/−^, and HCT116 p53^−/−^ cells treated with 6-SFN or JQ1 over a range of concentrations for 48 h. Mean ± SE; *n* = 3 biological replicates.

Constitutive *ERCC2* mRNA expression was high in colon cancer cells (SW480, HCT116) compared to normal colon epithelial cells (CCD841), as assessed by RT-qPCR (Figure 4A, *p* < 0.001). At 48 h, RT-qPCR analyses revealed that *ERCC2* mRNA levels were reduced highly effectively by 6-SFN + JQ1 in HCT116 and SW480 cells (Figure 4B and Figure S2A). In the corresponding cell lysates, loss of ERCC2 protein expression was confirmed by immunoblotting (IB) experiments, although high pH2AX and pRPA32 levels indicative of increased DNA damage were not confined solely to the 6-SFN + JQ1 group (Figure 4C and Figure S2B). Key molecular readouts in cells treated with 6-SFN + JQ1 included reduced Wnt/β-catenin signaling (MMP7, c-Myc), increased apoptosis (cleaved PARP and caspase-3), and induction of p53 and p21^WAF1^ (Figure 4C). Based on the latter observations, cell viability assays next were performed in p21 null and p53 null human colon cancer cells. Compared to the parental line, HCT116^p21−/−^ and HCT116^p53−/−^ cells responded similarly to 6-SFN and JQ1 treatment, whereas CCD841 normal colonic epithelial cells were more resistant to the test agents (Figure 4D,E). We concluded that p21 and p53 induction might contribute to cell cycle arrest [16,17], but these proteins are unlikely to be major players in the DNA damage response or apoptosis induction under the conditions used.

### 2.5. HDAC3 Inhibitor Plus BET Degrader Co-Treatment as Second-Generation Epigenetic Therapy

**Figure 5:**
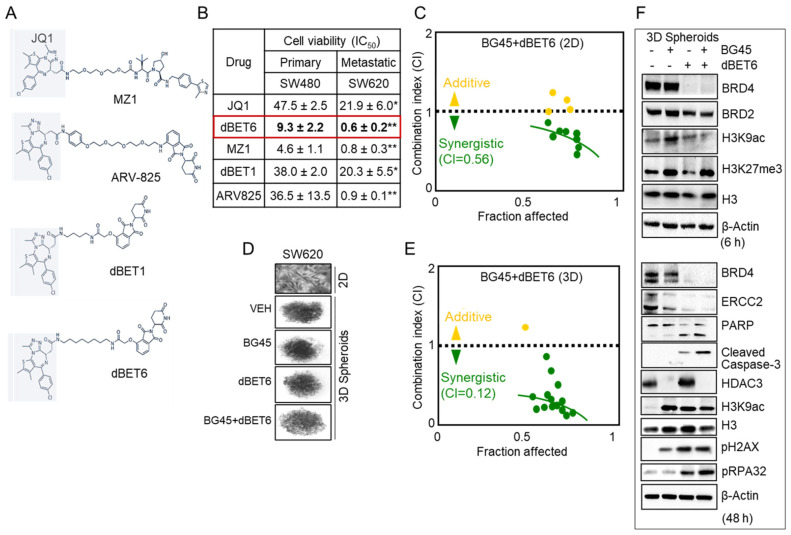
Second-generation deacetylase plus BET inhibition. (**A**) Chemical structures and (**B**) 50% growth inhibition (IC_50_) values in colon cancer cells treated with bromodomain and extraterminal domain (BET) inhibitors for 48 h; mean ± SE * *p* < 0.05, ** *p* < 0.01 (metastatic vs. primary). (**C**) Combination index (CI) data for SW620 cells in 2D monolayers treated with BG45 + dBET6, as in panel B. (**D**) Representative images of SW620 3D spheroids on day 3 after seeding 5000 cells/well. (**E**) CI data plotted for SW620 3D cells treated as in C. (**F**) Immunoblot analysis after SW620 3D spheroids were treated with Vehicle, BG45 (50 µM), dBET6 (1.56 µM), or BG45 + dBET6 for 6 and 48 h. β-Actin served as a loading control.

To identify epigenetic combinations with the potential for greater potency toward metastatic CRC, we evaluated several BET-degrader PROTACs (Figure 5A) and compared the cytotoxicity in SW480 non-metastatic vs. SW620 metastatic-lineage colon cancer cells. Based on the 50% inhibitory concentration (IC_50_) values, JQ1 and the PROTACs were significantly more effective in SW620 cells than in SW480 cells (Figure 5B, *p* < 0.05 or *p* < 0.01), with dBET6 being the most potent inhibitor. Subsequently, we combined dBET6 with the HDAC3-selective inhibitor BG45. The combination of BG45 + dBET6 exhibited synergy in SW620 cells (CI = 0.56, Figure 5C), which was markedly improved when SW620 cells were cultured as 3D spheroids (CI = 0.12, Figure 5D,E), indicating strong synergy. Immunoblotting of 3D cell lysates harvested after 6 h of treatment revealed early loss of BRD4 in the dBET6 and BG45 + dBET6 treatments, while histone acetylation and methylation changes were observed in the BG45 and BG45 + dBET6 treatments (Figure 5F, top). At 48 h, ERCC2 levels were reduced by the various treatments coinciding with a marked increase in the DNA damage marker pH2AX and enhanced pRPA32 indicating enhanced replication stress due to dBET6 and BG45 + dBET6 combination treatment. In addition, there was decreased BRD4 and HDAC3, increased histone acetylation, and cleaved PARP and caspase-3 indicative of apoptosis (Figure 5F, bottom). Notably, CCD841 normal colonic epithelial cells were much less susceptible to BG45 and dBET6 concentrations up to 50 μM (Figure S3).

### 2.6. Antitumor Activity of dBET6 + BG45 in SW620 Xenografts

**Figure 6:**
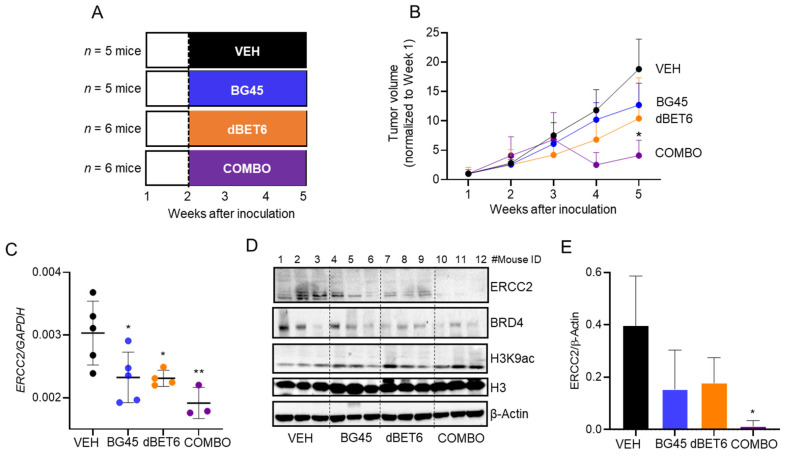
Antitumor activity of dBET6 + BG45 in SW620 xenografts. (**A**) Nude mice (5–6 animals/group) received vehicle (VEH), BG45 (50 mg/kg), dBET6 (7.5 mg/kg), or BG45 + dBET6 by *i.p.* injection, 3 times per week for 3 weeks. (**B**) Tumor volume changes over time following the inoculation of SW620 cells into nude mice. (**C**) *ERCC2* mRNA expression in xenografts at the end of the study, determined by RT-qPCR and normalized to *GAPDH*. (**D**) Immunoblotting of proteins as indicated, with histone H3 and β-actin as loading controls. (**E**) Quantification of ERCC2 protein expression from immunoblots, as described in Materials and Methods. In panels (**B**,**C**,**E**), mean ± SE, * *p* < 0.05, ** *p* < 0.01 vs. VEH alone. ‘Combo’ = combination drug treatment.

To examine the in vivo antitumor activity of BG45 + dBET6, we implanted SW620 cells into athymic nude mice (Figure 6A). Animals were treated thrice per week with vehicle, BG45 (50 mg/kg, *p.o.*), dBET6 (7.5 mg/kg, intraperitoneal (*i.p.*)), or BG45 + dBET6 in combination, starting one week after inoculation of mice with the human cancer cells (Figure 6A). At the end of the experiment, tumor outcomes were normalized to the initial volumes before the start of treatment; BG45 + dBET6 suppressed tumor growth significantly, the antitumor efficacy exceeding the inhibition observed for BG45 or dBET6 alone (Figure 6B, *p* < 0.05). By RT-qPCR analysis (Figure 6C), xenografts had reduced expression of *ERCC2* after BG45 or dBET6 treatment (*p* < 0.05), and there was greater inhibition by BG45 + dBET6 combined (*p* < 0.01). Loss of ERCC2 protein expression also was detected by IB analysis, especially for the combination treatment (Figure 6D,E, *p* < 0.05), along with decreased BRD4 and increased H3K9ac, compared with vehicle controls.

## 3. Discussion

A fundamentally important concept in cancer etiology is that the DNA damage response is initiated in the context of chromatin. There is a growing awareness of the intimate crosstalk between DNA repair and epigenetic regulatory mechanisms [18]. For example, the acetylation status of histone and non-histone proteins is governed by the opposing activities of histone acetyltransferase (HAT) and HDAC enzymes [19]. Acetylation also activates BRD proteins that localize at DNA breaks and promote chromatin remodeling to facilitate repair activities in the vicinity of the damage [20,21]. Thus, it is not surprising that combining HDAC and BRD inhibition can result in synergistic outcomes in cell-based assays (Figure 1A,B) and in preclinical models (Figure 1C,D). There is much interest in combination approaches that target epigenetic readers/writers/erasers, and in the current investigation, deacetylase plus bromodomain inhibition prioritized ERCC2 (Figure 2), with potential prognostic relevance for CRC patients (Figure 3).

Previously, we proposed that food-derived bioactives that affect the epigenome also might trigger DNA damage and repair responses [18]. The anticancer agent SFN and the more potent structurally-related dietary isothiocyanates (6-SFN and 9-SFN) inhibited HDAC activity and DNA damage/repair pathways in human colon cancer cells, but not in normal cells, by targeting CtBP-interacting protein (CtIP), which is a key player in homologous recombination (HR) [15]. Additionally, SFN was shown to disrupt protein–protein interactions of the HDAC3-regulated Wnt coactivator CCAR2 [13], which is a master regulator of HR and non-homologous end joining (NHEJ) [22,23]. Moreover, potent novel SFN and 6-SFN analogs that modified HAT and HDAC activities also attenuated HR/NHEJ repair mechanisms in colon cancer cells, providing a potential new avenue for chemosensitization [14]. 

We combined HDAC-specific inhibition and bromodomain inhibition with transcriptomics, via RNA-seq analyses. This strategy prioritized *ERCC2*, which is a key player in the NER pathway, providing further insights into the ‘cooperativity/synergy’ that exists between HDAC and BRD inhibitors. The precise mechanistic basis for this cooperativity remains to be fully elucidated; however, 6-SFN is a more potent HDAC inhibitor than SFN [15], and like SFN, 6-SFN might interact synergistically with JQ1 at the level of gene transcription. Ongoing ChIP assays, as previously reported for SFN + JQ1 with *MYC* [13], will determine whether HDAC3 and/or BRD4 binding on the *ERCC2* promoter and/or enhancer regions are inhibited by the combination of 6-SFN + JQ1. If indeed BRD4 levels are inhibited on *ERCC2*, it will be interesting to ascertain whether 6-SFN + JQ1 shifts the pool of acetyl readers in favor of BRD9-regulated genes, providing comparable mechanistic insights observed previously with SFN + JQ1 in combination [13].

To assess whether endogenous ERCC2 protein expression levels might account for the metastatic cell line SW620 being more sensitive to PROTACs than the SW480 parental counterpart (Figure 5B), we immunoblotted for ERCC2 in a panel of colon cancer and normal colonic epithelial cell lines (Figure S4). Higher ERCC2 expression was detected in cancer versus normal, as observed before (Figure 4A), but there were no obvious differences in ERCC2 expression between SW620 and SW480 (Figure S4). Because CtIP and CCAR2 were previously identified as mechanistic targets for SFN and SFN + JQ1, respectively, we used CRISPR/Cas9 in HCT116 colon cancer cells and observed markedly higher basal ERCC2 expression in CtIP^–/–^ and CCAR2^–/–^ cell lines compared to the parental cell line (Figure S5). Thus, mechanisms that also effectively downregulate ERCC2 might provide a ‘tipping point’ in colon cancer cells, being unable to mount an effective DNA damage response, and triggering apoptotic or alternative cell-death pathways. In other words, if HR + NHEJ pathways are inactivated by SFN analogs, the compensatory NER pathway requiring ERCC2 would be further compromised due to bromodomain inhibition, leading to a cellular crisis in cancer cells (Figure S6), unlike in normal colonic epithelial cells that are less susceptible to the drug treatments (Figure S3). Dose–response studies also indicated that BRD inhibition is vital in the downregulation of ERCC2 expression (Figure S7A). Notably, the ablation of ERCC2 using small interfering RNA (siRNA) in the metastatic cell line SW620 (Figure S7B) does not by itself increase the DNA damage marker, pH2AX. However, treatment with BG45 + dBET6 induced both pH2AX and further decreased ERCC2 levels, suggesting a dual mechanism of action, to be further validated using NER assays that measure DNA repair activity [24].

A critical challenge in the clinical translation of effective new anticancer agents is directing their activities preferentially toward tumor cells, while leaving healthy tissue less affected. The present investigation showed that higher *ERCC2* expression was associated with poor patient survival (Figure 3A), and ERCC2 protein levels can be markedly higher in cancer vs. normal tissue (Figure 3B), implying that drugs that target ERCC2 for downregulation are likely to have broader safety and therapeutic efficacy. Here, we showed that combining HDAC and BRD inhibition reduced the viability of colon cancer cells markedly, whereas normal colonic epithelial cells were more resistant (Figure 4D,E and Figure S3), providing an avenue for selective toxicity mechanisms via differential drug uptake or metabolism [14].

The current report also investigated the antitumor effects of second-generation BET degrader (dBET6) and HDAC3-specific inhibitor (BG45) agents in monolayers, spheroids, and xenografts (Figure 5 and Figure 6). Notably, BG45 + dBET6 exhibited ~4–5-fold higher potency in spheroids than in conventional monolayers (Figure 5), with the former assay conditions more closely resembling the tumor environment [25]. This was recapitulated in the SW620 xenograft model, with BG45 + dBET6 demonstrating significant antitumor activity, whereas the individual agents were less effective (Figure 6). These findings provide further support for the combined targeting of HDAC3 [26] and BRD4 as a mechanistic approach to precision epigenetic therapy. Interestingly, several unique *ERCC2* mutations were identified, with an overall frequency of 1.33% in CRC patients, from The Cancer Genome Atlas (TCGA) database (data not shown). This could be a pertinent consideration in future studies that assess epigenetic combinations in the context of chemosensitization to platinum-based therapy [24]. Finally, a future goal will be the identification of PROTAC-like bioactives from natural sources, as lead molecules that preferentially target HDAC3 and/or BRD4 for protein degradation, thereby enhancing chemosensitization in clinical trials [27].

## 4. Materials and Methods

### 4.1. Cells and Treatments

Human colon cancer cells (HCT116, SW480, SW620) and non-transformed colonic epithelial cells (CCD841) were purchased from ATCC and used within 10–15 passages from receipt. Each cell line was validated to be of human origin, with no mammalian interspecies contamination, and with the correct genetic profile based on allele-specific markers (Idexx Radil, Columbia, MO, USA) [28,29]. HCT116^p21−/−^ and HCT116^p53−/−^ cells were courtesy of Dr. Bert Vogelstein (Johns Hopkins University, Baltimore, MD, USA). Cells were cultured in McCoy’s 5A media or Eagle’s Minimum Essential Medium (EMEM) (Invitrogen), supplemented with 10% Fetal Bovine Serum (FBS) and 1% penicillin/streptomycin, at 37 °C in a humidified chamber with 5% CO_2_. The test agents JQ1 and BET degraders were purchased from MedChem Express (Monmouth Junction, NJ, USA), whereas 6-SFN was from LKT Laboratories (St. Paul, MN, USA). Stock solutions were made in DMSO, stored at −20 °C and thawed for single use before each experiment. Cells were treated with test agents 48 h after seeding, unless mentioned otherwise. In some experiments, additional cell lines, also obtained from ATCC, were used to examine endogenous levels of ERCC2, namely SW48, Caco2, HT29, LoVo, and HuTu80 (Figure S4).

### 4.2. Antiproliferation Assays

#### 4.2.1. Monolayers

Cell viability was determined using the MTT (3-(4,5-dimethylthiazol-2-yl)-2,5-diphenyltetrazolium bromide) assay, as reported previously [13,14,15]. Cells in the exponential growth phase were plated at a cell density of 5000 cells per well in 96-well tissue culture plates. After attachment overnight, cells were treated with compounds for 48 h at the concentrations indicated in the figures. MTT solution (20 μL) was added to each well of the 96-well plate and incubated for 4 h at room temperature. The absorbance was measured at 570 nm using a Cytation5 microplate reader (BioTek, Winooski, VT, USA). The combination index (CI) was calculated after treatment of cells with different drug doses and combinations for 48 h, and cell viability was measured by the MTT assay, as mentioned above. Subsequently, CI values were calculated using CompuSyn™ software, as previously reported [13]. In some experiments, the colony formation ability of HCT116 cells was assessed using reported methodologies [30].

#### 4.2.2. Spheroids

Nanoshuttle-PL magnetic nanoparticles (Greiner Bio-One, Monroe, NC, USA) were used for generating 3D spheroids of human colon cancer cells. Nanoparticles (1 µL Nanoshuttle/1 × 10^4^ cells) were added to SW620 cells and mixed gently, followed by centrifugation at 100× *g* for 5 min. Nanoparticle-bound cell pellets were resuspended, and the process was repeated twice. Magnetized cells were plated at a cell density of 1 × 10^4^ cells per well in a 96-well cell culture microclear blackplate and a cylindrical magnet facilitated spheroid formation at the bottom of the multiwell plate, replicating cell–cell interactions in three-dimensions [31,32]. Spheroids were treated with test agents for 48 h followed by assessment of the CI values, as described above. 

### 4.3. RNA Analyses 

Real-time quantitative PCR (RT-qPCR) was conducted as previously reported [13,33]. RNA was extracted from cell pellets and tumor samples using a NucleoSpin kit (Macherey-Nagel, Bethlehem, PA, USA), with quantification via a Cytation5 microplate reader. Reverse-transcription was performed utilizing SuperScript III (ThermoFisher, Waltham, MA, USA). Gene expression was quantified by qPCR in a 10-µL reaction volume, consisting of cDNAs, SYBR green dye (Genesee Scientific, San Diego, CA, USA), and gene-specific primers, in a LightCycler 480 II (Roche, Indianapolis, IN, USA). Each sample was subjected to three independent experiments, and quantification was based on the Ct value normalized to the housekeeping gene, as reported [13,33]. Murine and human gene-specific primers were as follows: rat *Ercc2:* 5′-ATGGGCTGCTGGTCTACTTC-3′ (F), 5′-TCCAGTGGATAAGCCCGTTG-3′ (R); rat *Glyceraldehyde-3-phosphate dehydrogenase* (*Gapdh)*: 5′-ATGGGAGTTGCTGTTGAAGTC-3′ (F), 5′-CCGAGGGCCCACTAAAGG-3′ (R); human *ERCC2*: 5′-CCTACATGCGGGAGCTCAAA-3′ (F), 5′-CAGCGGATATGCTCTCTGGT-3′ (R); human *GAPDH:* 5′-GACAGTCAGCCGCATCTTCT-3′ (F), 5′-GCGCCCAATACGACCAAATC-3′ (R). As reference genes used for the normalization of RT-qPCR data can sometimes vary under different experimental conditions [34], we verified that Ct values of human *GAPDH* and murine *Gapdh* expression were stable in our experiments.

RNA-sequencing (RNA-seq) and bioinformatics analyses were performed as reported [13,33], for adenomatous colon polyps from the Pirc model [35]. Library preparation via a NEBNext Ultra Directional RNA Library Prep Kit was followed by Illumina sequencing on a NextSeq 500/550 instrument (Illumina, San Diego, CA, USA). Quality control for fastq files was checked using Fastqc (V0.11.5, https://www.bioinformatics.babraham.ac.uk/projects/fastqc/). Pair-ended reads were mapped to human hg19 genome using Tophat (V2.1.1, https://github.com/infphilo/tophat), and uniquely mapped reads were extracted using SAMtools (V1.5, http://samtools.sourceforge.net/) as inputs for differentially expressed genes (DEGs). Cufflinks (V2.2.1, http://cole-trapnell-lab.github.io/cufflinks) was used to assemble the transcriptome using RefSeq annotation file to quantify gene expression level with reads per kb per million (FPKM). DEGs were identified using cuffdiff (V2.2.1, http://cole-trapnell-lab.github.io/cufflinks) with False Discovery Rate (FDR) ≤0.05 and absolute log2 fold change ≥1. Principle Component Analysis (PCA) used Bioconductor DESeq2 and heatmaps were generated via Ggplot2 (http://bioconductor.org/packages/release/bioc/html/DESeq2.html). All repositories were accessed between March and August 2017, and finally on 24 September 2020.

### 4.4. Immunoblotting

Immunoblotting (IB) used published procedures for whole cell lysates and tissue lysates [13,14,15]. Proteins (20 µg/lane) were separated by SDS-PAGE on 4–12% Bis-Tris gel (NuPAGE, Invitrogen, CA, USA) and transferred to nitrocellulose membranes (Invitrogen, CA, USA). Membranes were saturated with 2% Bovine Serum Albumin (BSA) for 1 h, followed by overnight incubation at 4 °C with primary antibodies for ERCC2 (GeneTex, Irvine, CA, USA, #GTX66267), cell cycle and apoptosis regulator protein 2 (CCAR2, Bethyl Laboratories, Montgomery, TX, USA, #8300-434A), HDAC3 (Santa Cruz, Dallas, TX, USA, #11417), pH2AX Ser139 (Santa Cruz, Dallas, TX, USA, #101696), pRPA32 S4/S8 (Bethyl Laboratories, Montgomery, TX, USA, #A300-245A), histone H3K27me3 (Active-Motif, Carlsbad, CA, #39156), H3K9ac (Sigma-Aldrich, St. Louis, MO, USA, #07-352), and β-Actin (Sigma-Aldrich, St. Louis, MO, USA, #A5441). Antibodies for p21 (#2947), p53 (#9282S), BRD4 (#E2A7X), BRD2 (#D89B4), H3 (#D2B12), c-Myc (#D3N8F), MMP7 (#3801S), poly(ADP-ribose)polymerase (PARP) (#9542), and cleaved caspase-3 (#9661) were from Cell Signaling (Danvers, MA, USA). After washing, membranes were incubated with horseradish peroxidase-conjugated secondary antibodies for 1 h. Bands were visualized using Western Lightning Plus-ECL Enhanced Chemiluminescence Substrate (Perkin Elmer, Waltham, MA, USA) and detected using a ChemiDoc MP Imaging System (Bio-Rad, Hercules, CA, USA). 

### 4.5. Preclinical Experiments

All studies were approved by the Institutional Animal Care and Use Committee. In rat experiments, Pirc males at 5 months of age were assigned to study groups (3–4/group). Rats were then treated for 2 months with test agents, as follows: 6-SFN, 10 mg/kg body weight via daily oral gavage (*p.o.*); JQ1, 10 mg/kg body weight via twice weekly intraperitoneal (*i.p.*) injection; 6-SFN + JQ1 at the doses of the individual compounds, or vehicle. At the end of the study, colon polyps were enumerated, and tissues were bio-banked for molecular analyses, as reported [13,33,36].

Xenograft experiments were done as previously described [13]. Briefly, SW620 cells (5 × 10^6^) were injected into either flank of male athymic nude mice (Envigo, Indianapolis, IN, USA). After a week, animals were randomized to treatment groups, as follows (*n* = 5 mice/group): BG45, 50 mg/kg body weight; dBET6, 7.5 mg/kg body weight, thrice weekly *i.p.* injections for 4 weeks; BG45 + dBET6, at the doses of the individual compounds, or vehicle. Tumor volumes were measured twice per week using Vernier calipers. 

### 4.6. Statistical Analyses

Results are representative of findings from at least three independent experiments, expressed as mean ± SE, unless indicated otherwise. Multiple groups were subjected to analysis of variance (ANOVA) and the Bonferroni test in GraphPad Prism v5.04. Statistical significance was indicated in the figures as follows: * *p* < 0.05, ** *p* < 0.01, *** *p* < 001, and **** *p* < 0.0001.

## 5. Conclusions

The current investigation with JQ1 + 6-SFN defined ERCC2 as a key mechanistic target in human colon cancer cells, with the potential to impact overall survival in CRC patients. Second-generation BET degrader plus HDAC3-specific inhibition (dBET6 + BG45) was highly effective in metastasis-lineage colon cancer cells, exhibiting marked downregulation of ERCC2 in monolayers, spheroids, and xenografts, coinciding with antitumor outcomes in vivo. We conclude that further studies are warranted into the clinical relevance of ERCC2, a key cellular DNA repair protein, as a potential biomarker and therapeutic target for cancer interception and epigenetic combination therapy. 

## Data Availability

Data available in a publicly accessible repository.

## Supplementary Materials

The following are available online at https://www.mdpi.com/2072-6694/13/6/1438/s1, Figure S1: Colony formation of human colon cancer cells treated with 6-SFN, JQ1, or 6-SFN+JQ1 over a range of concentrations for 48 h. Mean ± SE, *n* = 3 biological replicates. Figure S2: *ERCC2* expression analyzed by RT-qPCR and immunoblotting in SW480 cells treated with drugs for 48 h. β-Actin, loading control, ** *p* < 0.01, *** *p* < 0.001. Figure S3: Viability of normal (CCD841, colonic epithelial cells) and cancer (SW620) cells treated with (A) BG45 or (B) dBET6 over a range of concentrations for 48 h. Mean ± SE, *n* = 3 biological replicates. Figure S4: Immunoblotting of cell lysates from colon cancer and normal cell lines. Figure S5: Immunoblotting of cell lysates from (lane 1) HCT116 parental, (lane 2) HCT116 CCAR2-null, and (lane 3) HCT116 CtIP-null cells, using CRISPR/Cas9 for genome editing. Arrow, cleaved PARP. Figure S6: Working model of deacetylase plus bromodomain inhibition impacting DNA repair and cancer cell death. Figure S7: Immunoblotting of SW620 cell lysates following (B) ERCC2 siRNA treatment and (A) different doses of BRD inhibitors (dBET6 and JQ1) after 48 h of treatment. β-Actin, loading control.
