# Supplementary material for: Deacetylase Plus Bromodomain Inhibition Downregulates ERCC2 and Suppresses the Growth of Metastatic Colon Cancer Cells
Source: Cancers (Basel). 2021 Mar 22;13(6):1438. doi: 10.3390/cancers13061438 (PMC8004213; doi:10.3390/cancers13061438)
Supplement: Supplementary file 1 [file cancers-13-01438-s001.pdf]

Supplementary Material: Deacetylase Plus Bromodomain Inhibition Downregulates ERCC2 and Suppresses the Growth of Metastatic Colon Cancer Cells

Sabeeta Kapoor, Trace Gustafson, Mutian Zhang, Ying-Shiuan Chen, Jia Li, Nhung Nguyen, Jorge Enrique Tovar Perez, Wan Mohaiza Dashwood, Praveen Rajendran and Roderick H. Dashwood

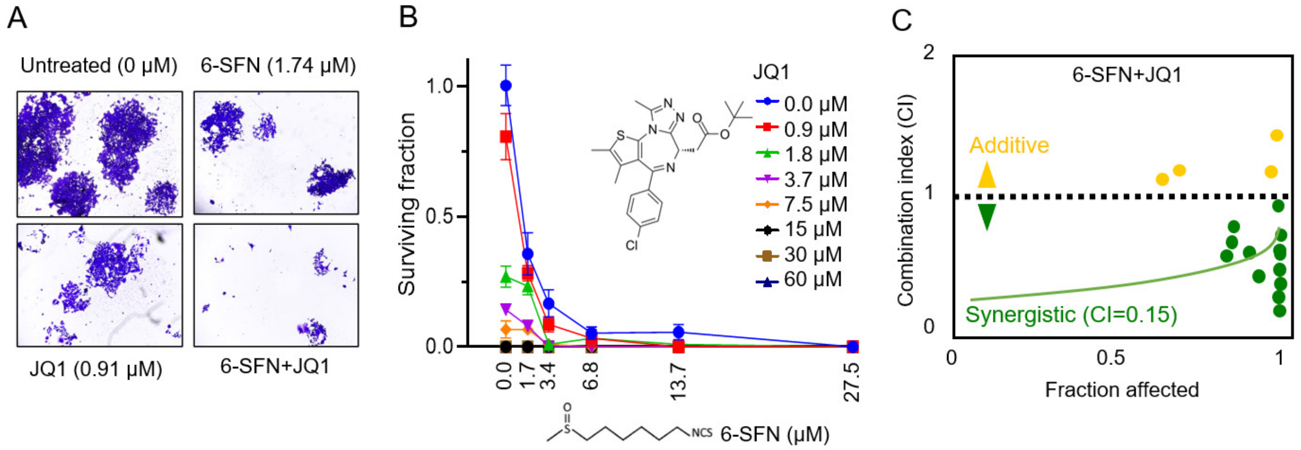

Figure S1. Colony formation of human colon cancer cells treated with 6-SFN, JQ1, or 6-SFN+JQ1 over a range of concentrations for 48 h. Mean ± SE, $n = 3$ biological replicates. (**A**) Representative microscopic images of HCT116 colonies stained using crystal violet, (**B**) Surviving fraction of colonies plotted against different drug concentrations, and (**C**) Combination index (CI) data from the colony formation assay in panel B; CI <1.0 indicates synergy; the lowest CI value of 0.15 indicated highly synergistic inhibition for 6-SFN+JQ1 in combination.

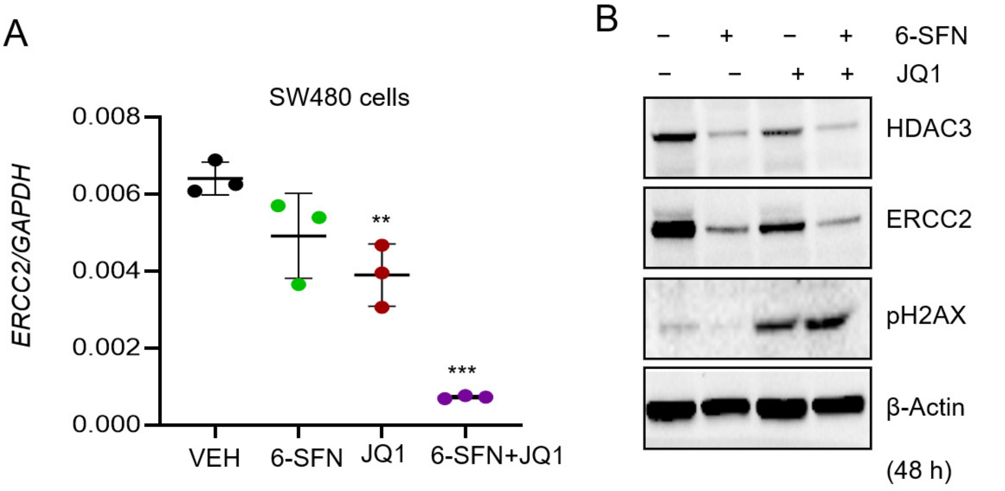

Figure S2. *ERCC2* expression analyzed by (**A**) RT-qPCR and (**B**) immunoblotting in SW480 cells treated with drugs for 48 h. β-Actin, loading control, $^{**}p < 0.01$, $^{***}p < 0.001$.

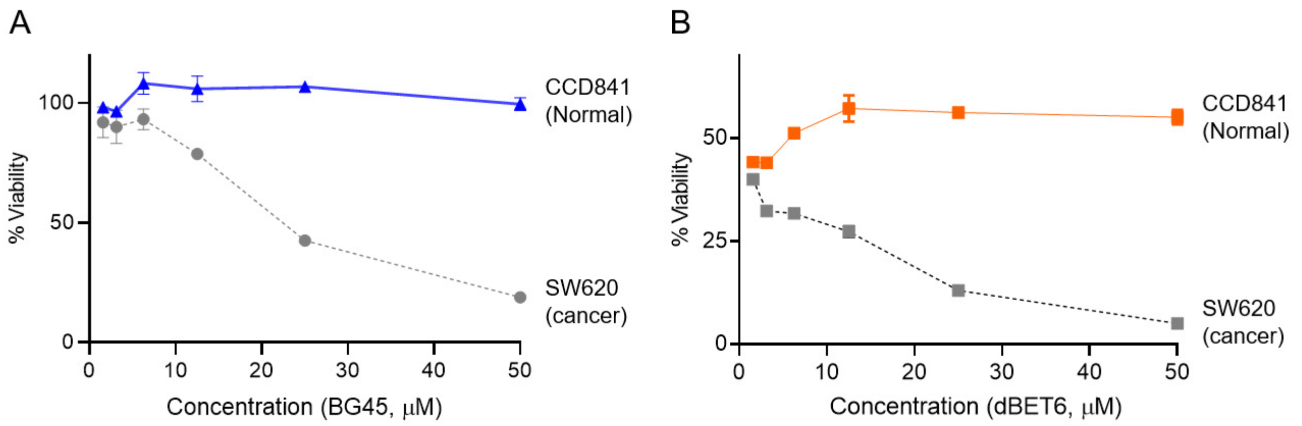

Figure S3. Viability of normal (CCD841, colonic epithelial cells) and cancer (SW620) cells treated with (**A**) BG45 or (**B**) dBET6 over a range of concentrations for 48 h. Mean ± SE, n = 3 biological replicates.

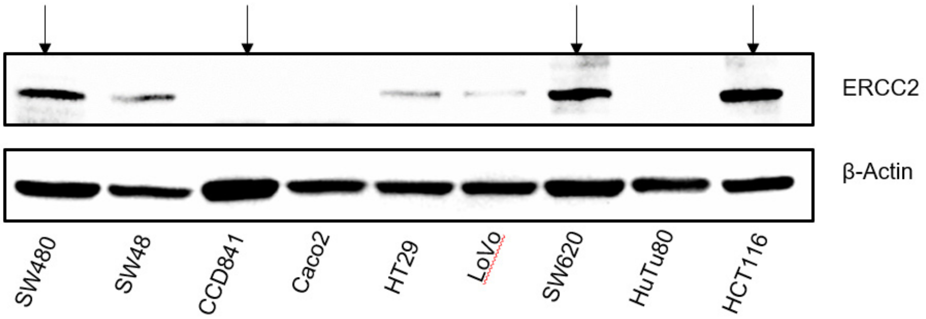

Figure S4. Immunoblotting of cell lysates from colon cancer and normal cell lines. Arrows indicate the four cell lines used in the study, including CCD841, the normal colonic epithelial cells. β-Actin, loading control.

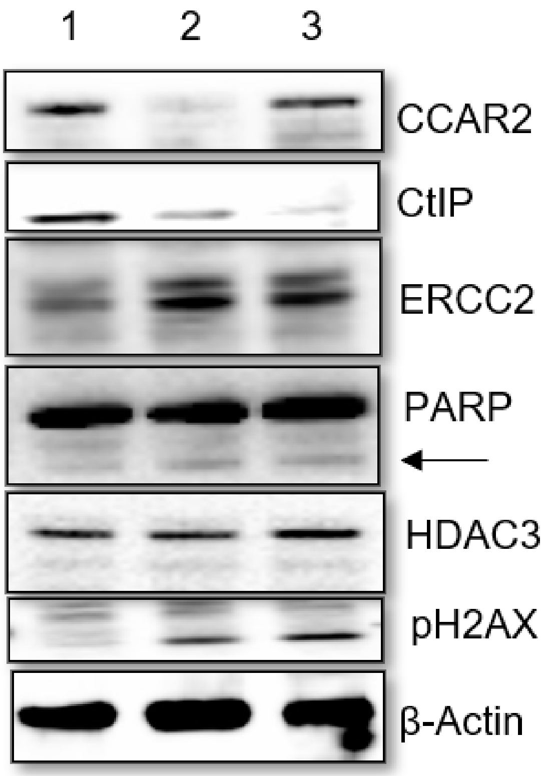

Figure S5. Immunoblotting of cell lysates from (lane 1) HCT116 parental, (lane 2) HCT116 CCAR2-null, and (lane 3) HCT116 CtIP-null cells, using CRISPR/Cas9 for genome editing. Arrow, cleaved PARP.

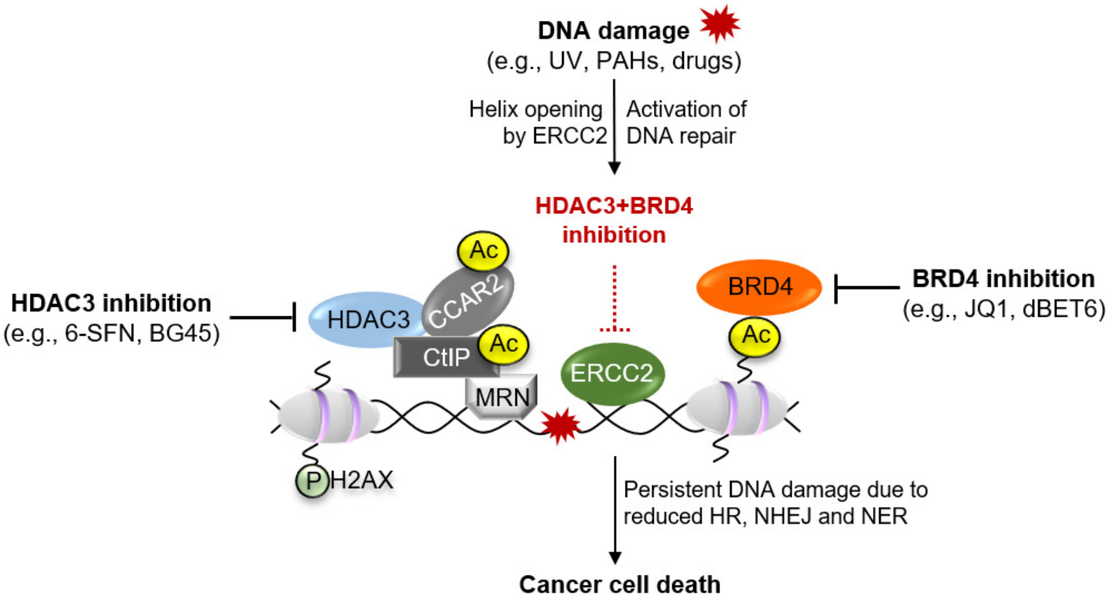

Figure S6. Working model of deacetylase plus bromodomain inhibition impacting DNA repair and cancer cell death. DNA damage caused by UV light, environmental polycyclic aromatic hydrocarbons (PAHs), and platinum-based chemotherapy drugs triggers increased pH2AX levels. The DNA helix is opened by Excision repair cross-complementation group 2 (ERCC2), initiating non-homologous end-joining (NHEJ), homologous recombination (HR) and/or nucleotide excision repair (NER). The Mre11/Rad50/NBS1 (MRN) complex is bound by HR repair factor CtBP-interacting protein (CtIP) and/or NHEJ 'master regulator' Cell cycle and apoptosis regulator protein 2 (CCAR2). Histone acetyltransferases and histone deacetylases (HDACs), such as HDAC3, regulate the acetylation status of histone and non-histone proteins in the vicinity,

including CtIP and CCAR2, which interact with acetyl 'readers' such as bromodomain-containing protein 4 (BRD4), leading to efficient DNA repair. Combined deacetylase plus bromodomain inhibition results in an initial downregulation of NHEJ + HR factors in colon cancer cells, and the attempted compensatory upregulation of NER proteins, such as ERCC2. However, as reported here for the first time, continued HDAC3 + BRD4 inhibition also impairs ERCC2 expression in the NER pathway (red dotted symbol), exacerbating the DNA damage and triggering apoptotic death in cancer cells. Ac, acetylation; P, phosphorylation.

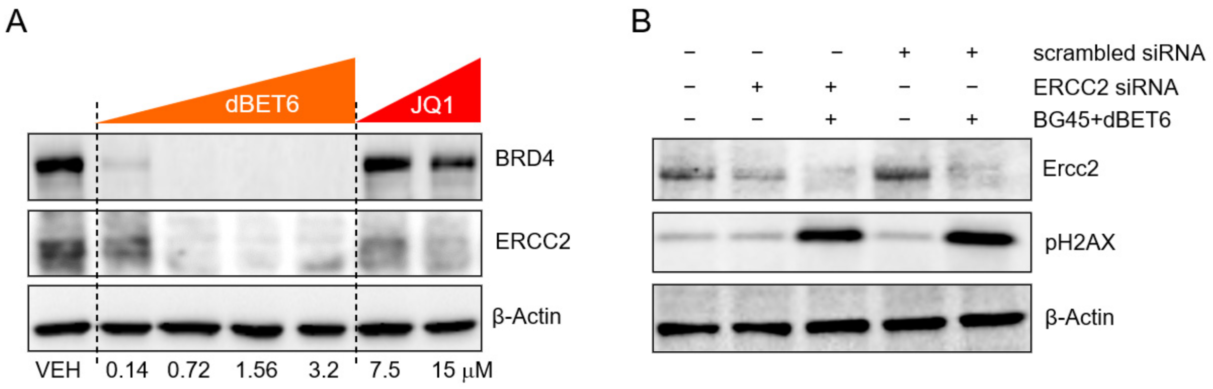

Figure S7. Immunoblotting of SW620 cell lysates following (**A**) different doses of BRD inhibitors (dBET6 and JQ1) after 48 h of treatment and (**B**) ERCC2 siRNA treatment. β-Actin, loading control.
